# Auditory thalamus dysfunction and pathophysiology in tinnitus: a predictive network hypothesis

**DOI:** 10.1007/s00429-021-02284-x

**Published:** 2021-05-02

**Authors:** Pia Brinkmann, Sonja A. Kotz, Jasper V. Smit, Marcus L. F. Janssen, Michael Schwartze

**Affiliations:** 1 Department of Neuropsychology and Psychopharmacology, University of Maastricht, Universiteitssingel 40, 6229 Maastricht, The Netherlands; 2 Department of Neuropsychology, Max Planck Institute for Human Cognitive and Brain Sciences, Leipzig, Germany; 3 Department of Ear Nose and Throat/Head and Neck Surgery, Zuyderland Medical Center, Sittard/Heerlen, the Netherlands; 4 Department of Clinical Neurophysiology, Maastricht University Medical Center, Maastricht, The Netherlands; 5 School for Mental Health and Neuroscience, Faculty of Health, Medicine and Life Sciences, Maastricht University, Maastricht, The Netherlands

**Keywords:** Tinnitus, Medial geniculate nucleus, MGB, Prediction, Temporal processing

## Abstract

Tinnitus is the perception of a ‘ringing’ sound without an acoustic source. It is generally accepted that tinnitus develops after peripheral hearing loss and is associated with altered auditory processing. The thalamus is a crucial relay in the underlying pathways that actively shapes processing of auditory signals before the respective information reaches the cerebral cortex. Here, we review animal and human evidence to define thalamic function in tinnitus. Overall increased spontaneous firing patterns and altered coherence between the thalamic medial geniculate body (MGB) and auditory cortices is observed in animal models of tinnitus. It is likely that the functional connectivity between the MGB and primary and secondary auditory cortices is reduced in humans. Conversely, there are indications for increased connectivity between the MGB and several areas in the cingulate cortex and posterior cerebellar regions, as well as variability in connectivity between the MGB and frontal areas regarding laterality and orientation in the inferior, medial and superior frontal gyrus. We suggest that these changes affect adaptive sensory gating of temporal and spectral sound features along the auditory pathway, reflecting dysfunction in an extensive thalamo-cortical network implicated in predictive temporal adaptation to the auditory environment. Modulation of temporal characteristics of input signals might hence factor into a *thalamo-cortical dysrhythmia* profile of tinnitus, but could ultimately also establish new directions for treatment options for persons with tinnitus.

## Introduction

Tinnitus is frequently described as hearing a sound without an external source or as a ringing in the ears (Baguley et al. 2013). The prevalence of tinnitus ranges from 10 to 15% in the general population, and in 1–2% it severely interferes with the affected person’s daily life (Langguth et al. 2013; McCormack et al. 2016; Schlee et al. 2017). Severe forms of tinnitus exert a particular negative impact on the quality of life, with symptoms of depression, anxiety, sleep disturbances, concentration difficulties, or reduced cognitive efficiency (Hallam et al. 2004; Langguth 2011). Consequently, tinnitus has direct societal impact, as reflected in high healthcare costs and loss of productivity (Maes et al. 2013). Currently, there is no curative evidence-based therapy for tinnitus, i.e., although drug targets, cognitive, behavioral, and neuromodulative interventions have been put forward, there is a lack of randomized controlled trials confirming effective tinnitus treatment (Kleinjung and Langguth 2020).

Over the past two decades, general interest in tinnitus has rapidly grown as part and parcel of new hypotheses about tinnitus pathophysiology (Moller et al. 2015; Roberts and Salvi 2019). Reflecting parallel advances in neuroimaging methodology, the general focus shifted from otology to neuronal correlates of tinnitus (Langguth et al. 2013). Although there is no strong consensus, it is generally assumed that hearing loss precedes the development of tinnitus. Consequently, changes along the classical and non-classical auditory pathway, expressed in alterations of spontaneous firing activities, neural synchronization, or tonotopic organization are possible key elements of tinnitus pathogenesis (Elgoyhen et al. 2015).

**Fig. 1:**
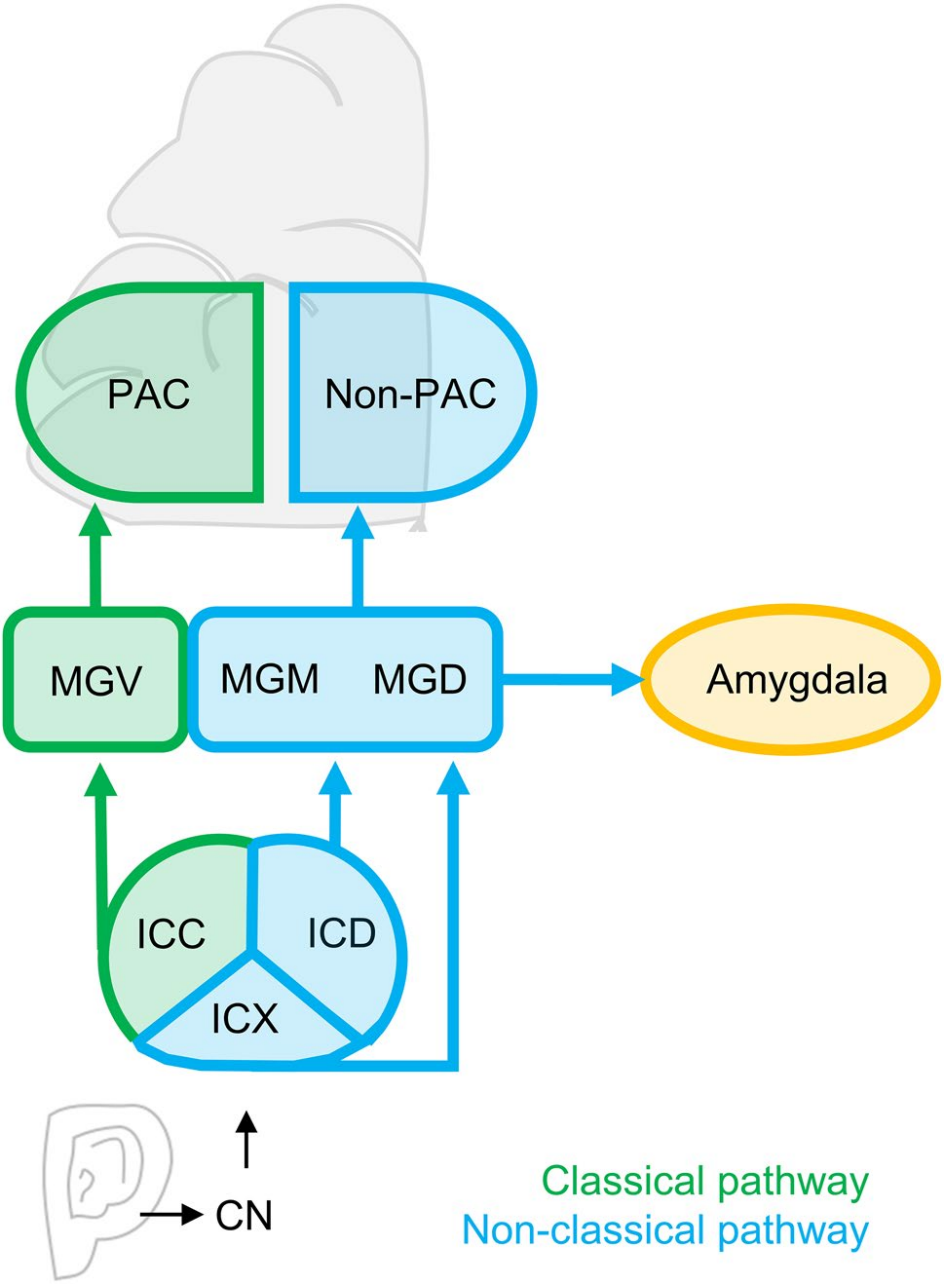
Schematic and simplified representation of the classical and non-classical ascending auditory pathway. Ascending auditory signal travels from the ears to primary and secondary auditory cortices, while taking two different pathways. *PAC* primary auditory cortex, *Non-PAC* non-primary auditory cortices, *CN* cochlear nucleus, *ICC* central inferior colliculus, *ICD* dorsal inferior colliculus, *ICX*, external inferior colliculus, *MGB* medial geniculate body, *MGD* dorsal MGB, *MGM* medial MGB, *MGV* ventral MGB

The classical ascending auditory pathway includes projections to mainly primary auditory regions, while non-classical auditory pathways have been described as extralemniscal, diffuse, or polysensory pathways that involve connections to non-primary auditory areas (Fig. 1) (Aitkin 1986; Graybiel 1972; Møller 2012). Next to neural correlates of tinnitus, current theories suggest maladaptive gating, increased central gain or altered neural thalamo-cortical coherence as factors underlying the development of tinnitus (De Ridder et al. 2015; Llinas et al. 1999; Norena 2011; Rauschecker et al. 2010). However, although the auditory thalamus, and in particular the medial geniculate body (MGB), is a mandatory relay station along the auditory pathway, its contribution to tinnitus pathology is often disregarded.

The MGB is part of the classical and non-classical auditory pathway, mediating the thalamo-cortical network involved in tinnitus. It actively shapes information processing between subcortical and cortical areas (Bartlett 2013; De Ridder et al. 2015; Llinas et al. 1999). Animal research provides first indications of successful tinnitus treatment by invasively stimulating the MGB in rats (van Zwieten et al. 2019b). The MGB should hence not only be considered a major gateway station for auditory signals transmitted to the cerebral cortex, but also as a crucial component in developing a better understanding of tinnitus pathology (Leaver et al. 2011; Moller 2003). Taking this perspective and starting with a review of thalamic contributions to auditory processing, we formulate a hypothesis of thalamic functioning in tinnitus pathology from a comparative perspective, integrating animal and human evidence. We propose that changes in thalamic functioning affect sensory gating at the level of the MGB, suggesting a dedicated timing and temporal prediction mechanism as an independent source of information and a potential tool for modulating the experience of tinnitus.

## Functional neuroanatomy of the medial geniculate body of the thalamus

To improve understanding of tinnitus and the role of the auditory thalamus in tinnitus pathophysiology, it is necessary to first consider the functional anatomy of the MGB. In general, the auditory pathway contains ascending and descending connections to auditory cortices and along its way, information is transformed and reorganized (Møller 2011; Oertel and Doupe 2013). Input travels through the ear, the cochlea (Fig. 1), the cochlear nuclei (CN) and the inferior colliculus (IC) before reaching the MGB (Oertel and Doupe 2013).

Originating from the IC, two ascending pathways, the classical and the non-classical auditory pathway innervate the MGB, primary (PAC) and non-primary auditory cortices (non-PAC), as well as limbic regions (Møller 2002; Pickles 2015). The IC can be divided into three distinct nuclei, the central part of the IC (ICC), the dorsal cortex of the IC (ICD), and the external nucleus of the IC (ICX). In the classical pathway, the ICC provides the main input to the ventral MGB (MGV; Table 1 for an overview).

**Table 1:** Overview of MGB subdivisions and their functionality

	Ventral MGB	Medial MGB	Dorsal MGB
Primary role in functional neuroanatomy	Auditory relay	Multisensory relay	Multisensory relay
Classical/non-classical auditory pathway	Classical	Non-classical	Non-classical
Input	Ipsilateral central nucleus of the IC	External IC, dorsal IC, central IC, lateral tegmentum, spinal cord, superior colliculus	Dorsal IC, tegmentum, sagulum, somatosensory system
Output	Ipsilateral primary auditory cortex (PAC)	Primary and secondary auditory cortices (PAC, non-PAC), lateral nucleus of the amygdala, non-auditory areas, striatum	Secondary auditory cortices (non-PAC, i.e., belt areas), lateral nucleus of the amygdala, non-auditory areas
Responses to sound	Sharp tuning curves, single-peaked, short tone latency, closely connected to the PAC	Heterogeneous tuning curves, spatial selectivity, short tone latency, fire at stimulus onset, associative learning responses (i.e., fear conditioning)	Wide, multipeaked tuning curves, short and long tone latency, sharp tuning curves, habituation and stimulus adaptation
Tonotopic organization	Yes	Yes (less than ventral MGB)	No
Tonotopic map	Low-to-high ( ventrolateral-dorsomedial)	NA	NA

*PAC* primary auditory cortex, *Non-PAC* secondary non-primary auditory cortex, *IC* inferior colliculus, *MGB* medial geniculate body

The MGV forms the “core” subdivision of the MGB. The MGV has a pronounced tonotopic organization, narrow tone frequency tuning, and exclusively responds to auditory input (Aitkin and Webster 1972; Bartlett 2013; Hackett et al. 2011). Fibers from the MGV primarily innervate the primary auditory cortex (Bartlett 2013).

The non-classical pathway processes input beyond the auditory domain and innervates limbic regions, such as the amygdala, next to primary and secondary auditory cortices (Bartlett 2013; Møller 2002). The ICD and the ICX provide input to the medial and dorsal subdivisions of the MGB in the non-classical pathway. The dorsal subdivision of the MGB (MGD) is not tonotopically organized (Bartlett 2013) and projects to the Non-PAC, the lateral nucleus of the amygdala and non-auditory areas (Bartlett 2013). The medial subdivision of the MGB (MGM) is the most heterogeneous part of the MGB. The tonotopic organization of the MGM is not as pronounced as in the MGV, tone frequency tuning is heterogeneous and neurons in the MGM respond not only to auditory, but also to visual and somatosensory input (Bartlett 2013; Hackett et al. 2011; Rouiller et al. 1989). Projections from the MGM terminate in primary and non-primary auditory cortices as well as the amygdala (Aitkin 1986; Bartlett 2013; Møller 2002). Moreover, all MGB subdivisions receive input from the reticular nucleus in the thalamus (TRN), which influences general excitability of neuronal activity in the MGB (Bartlett 2013; Møller 2002).

Consequently, classical and non-classical ascending auditory pathways contribute differently to the processing of auditory stimuli in the MGB and most likely to tinnitus pathophysiology. Due to the different input and output structures, the three subdivisions of the MGB may form three separate and parallel pathways to higher cortical auditory areas (Pickles 2015; Winer et al. 2005).

## Tonotopic map and sound level tuning in the MGB

As described above, the MGB is divided into different parts, the MGD, MGM and MGV. These parts have different neurophysiological properties and respond differently to external auditory stimuli. Frequency maps have been created in animal models by means of electrophysiological studies. Frequency tuning in the MGB is sharpest in the MGV in awake marmoset primates (Bartlett et al. 2011). Similar results were obtained in rats (Bordi and LeDoux 1994). Comparable to the MGV, neurons in the MGM have also been shown to respond in a narrow fashion in the anesthetized rat (Anderson and Linden 2011). An intermediate level of frequency tuning has been described for the MGD (Bartlett 2013). However, results are not consistent and fast habituation to repeated stimuli using isointensity tones in the MGD are described in Bordi and LeDoux (1994). The picture is even more complex as recent evidence suggests more variable tonotopic maps for neurons with multi-peaked frequency tuning curves (Gaucher et al. 2019).

Sound level tuning on the other hand, is monotonic, i.e., the sound intensity changes, while the frequency remains stable. Neurons exhibiting either a progressive increase or a progressive decrease in firing rates when intensity changes can be classified as monotonic. Non-monotonic responses are observed when firing rates increase with increasing tone intensity until a plateau is reached, after which firing rates decrease. Rouiller et al. (1983) investigated sound level tuning in the MGB in anesthetized cats and found that the majority of units exhibited non-monotonic responses.

Animal studies investigating the MGB predominantly rely on invasive techniques whereas in human studies non-invasive methods are predominant due to self-evident ethical reasons. This makes the identification and subsequent manipulations of the small (i.e., 5 × 4 × 5 mm, Winer et al. 1984) and densely clustered MGB nuclei intrinsically difficult. Technological advances such as ultra-high field functional neuroimaging (i.e., 7 T) allow creating precise tonotopic maps of the human MGB (Berlot et al. 2020; Mihai et al. 2019a; Moerel et al. 2015). These functional magnetic resonance imaging (fMRI) techniques allow high spatial resolution imaging, but depend on slow changes in blood oxygenation. Thus the signal depends on the vascular morphology of the relatively small MGB (Moerel et al. 2015). In humans, a low-to-high tonotopic map has been identified in the MGV in a ventrolateral-dorsomedial direction (Moerel et al. 2015). Moerel et al. (2015) further observed another, dorsomedial area with a preference for low frequency stimuli, located outside the MGV. Berlot et al. (2020) investigated the MGB tonotopy in persons with tinnitus and healthy controls, confirming a low–high–low frequency preference in the sagittal plane, comparable between groups. Tonotopic organization of the MGV and of the pars lateralis (PL) in the MGB in anesthetized cats has been found to also range from low-to-high in a latero-medial gradient (Aitkin and Webster 1972; Morel et al. 1987). Thus, mounting evidence supports a roughly similar low-to-high tonotopic organization in animals and humans in the MGV, validating the comparative usage of animal models.

### Representation of complex sounds

It is likely that artificially created sine tones do not entirely capture the functioning of the MGB when it perceives more complex auditory stimuli, such as vocalizations. In awake guinea pigs, the MGB has been shown to respond to amplitude-modulated (AM) and frequency-modulated (FM) sine tones as well as to natural calls (Creutzfeldt et al. 1980). Interestingly, the MGB responded to natural calls of the same and of other species, and its response was depicted in more detail in the MGB than in cortical cells, meaning that MGB units could differentiate between high modulation frequencies, while cortical cells could not (Creutzfeldt et al. 1980).

Discrimination of speech-like contrasts seems to occur at the level of the MGB, as observed in mismatch responses in the caudo-medial MGB of guinea pigs (Kraus et al. 1994). Cai et al. (2016) investigated whether young, old, awake, or anesthetized rats differentially process complex auditory stimuli in the MGB. They found that MGB cells in the old awake rat preferred regular predictable, vocalization-like signals, especially when increasing the difficulty in modulation frequency (Cai et al. 2016). In young rats, however, randomly presented modulated sequences were preferred (Cai et al. 2016). This suggests that with increasing age, top–down processes may enhance the processing of expected stimuli with the same formal structure at the level of the MGB. Accordingly, previous research shows that the MGB is not only tonotopically organized, but that it is closely involved in the representation of complex vocalizations across species and that its functioning may change across the life span (i.e., preferring predictable stimuli) (Amin et al. 2010; Cai et al. 2016; Huetz et al. 2009; Kraus et al. 1994).

Human research specifically targeting MGB activity in response to human vocalization and speech is rare (Mihai et al. 2019a, c). The MGB is active irrespective of content or loudness manipulations of speech sounds (von Kriegstein et al. 2008). Mihai et al. (2019a) assessed speech recognition abilities in the core subdivision of the auditory thalamus (i.e., MGV) and found behaviorally-relevant task dependent fMRI modulation of the left MGV. Furthermore, left MGV was found to be increasingly activated when participants had to recognize speech in noise compared to intelligible speech (Mihai et al. 2019b). Previously, the ventral intermediate nucleus (VIM) has been reported to respond to syntactic and semantic components in spoken language (Wahl et al. 2008). In addition, it has been proposed that the thalamus contributes to speech processing via its differential encoding of temporal and spectro-temporal information (Kotz and Schwartze 2010). Taken together, evidence across several species indicates that the MGB dynamically shapes simple tones and complex vocalizations before auditory sensations reach the cerebral cortex.

## Information processing in the MGB—intrinsic cell properties

To gain better understanding of information processing in the auditory thalamus and how these processes transform auditory information before it reaches the cortex, it is necessary to focus on specific electrophysiological properties of MGB neurons. Thalamic neurons respond to incoming information in either a burst or a tonic mode (Sherman and Guillery 2006). Thus, questions arise as to how the two firing modes (i.e., burst and tonic mode) emerge and how they shape auditory information processing.

Next to classical action potentials (i.e., single-spikes), low threshold spikes (LTS) are important voltage dependent conductance mechanisms for thalamic relay cells (Jahnsen and Llinas 1984b; Sherman and Guillery 2006). A LTS involves the membrane depolarization of T-type voltage-gated calcium channels (Jahnsen and Llinas 1984a, b), while classical action potentials are provoked by the opening of sodium (Na^+^) channels. The threshold to elicit a LTS is approximately 10 mV lower (i.e., more hyperpolarized) than for classical action potentials (Hu 1995; Jahnsen and Llinas 1984b; McCormick et al. 1991). In addition to the fact that T-type calcium channels act on more hyperpolarized membrane potentials, than sodium channels, T-type calcium channels are slower and need approximately 100 ms to switch between states of inactivation (Sherman and Guillery 2006).

Of specific interest for subsequent information processing in the MGB are the two different firing modes in response to LTSs. The state of the T-type calcium channels, determines the respective firing mode of the thalamic neurons (Ramcharan et al. 2000). Irrespective of the neuron type, thalamic neurons have been found to respond in either a tonic or a burst mode and also switch between these modes (Jahnsen and Llinas 1984b; McCormick et al. 1991; Sherman 2001; Sherman and Guillery 2006). The tonic mode has been described as preserving input linearity, whereas the burst mode acts as a ‘wake-up call’ to cortical targets (Ramcharan et al. 2000; Sherman 1996). Rhythmic burst firing has been primarily observed during sleep, potentially indicating reduced transmission of sensory information to the cortex (Domich et al. 1986; Sherman and Guillery 2006). However, it has been shown that burst firing is not limited to sleep and can be recorded from the thalamus of awake behaving macaque monkeys (Ramcharan et al. 2000). Information processing in burst mode has been suggested to be less detailed and less noisy, but also more efficient, as only infrequent ‘wake-up calls’ are processed (Sherman and Guillery 2006). Information processing in the tonic mode, however, maintains a more detailed representation of an input signal (i.e., more linearity).

When T-type channels are inactivated by membrane depolarization, the tonic mode is elicited (Ramcharan et al. 2000). To elicit burst firing, T-type calcium channels are activated from a hyperpolarized condition (Ramcharan et al. 2000). In mice, it was shown that switching between burst and tonic firing in MGV neurons partly underlies paired-pulse depression in thalamo-cortical neurons (Bayazitov et al. 2013). Bayazitov et al. (2013) employed an auditory paired-pulse paradigm (i.e., intra-pair-interval = 100–1000 ms, inter-pair interval = 500–10,000 ms) and found that thalamic neurons responded to the first tone of the pair with a burst, followed by a single-spike action potential. Furthermore, it was found that the point of switching between the burst firing—single-spike pattern to a single-spike—single-spike pattern in response to a stimulus pair occurs around an inter-pair-interval of 1000 ms, when applying intra-pair-intervals of 200–1000 ms (Bayazitov et al. 2013). These results indicate temporal sensitivity when switching between the tonic and the burst firing mode. A similar hypothesis has previously been formulated by Bartlett (2013), stating that in speech, where fast-changing temporal features are common, burst firing may encode the rhythmic dynamic of syllabic on- and offsets, while tonic firing may help discriminating between finer, more faint auditory signals. In addition, it has been suggested that burst mode patterns are more frequently encountered in MGD neurons and single-spike firing predominantly in MGV (Hu 1995), a pattern that has not been confirmed by Bartlett and Smith (1999). Thalamic cells thus fire in a burst or a tonic mode, which map onto non-linear and linear information processing, respectively. However, it is unclear how the different firing modes and spiking patterns may relate to tinnitus pathology.

## The MGB in tinnitus pathology

Animal models of tinnitus are frequently employed to systematically investigate the pathophysiology of tinnitus and the changes it causes along the auditory pathway, including the MGB. These models can be broadly divided into interrogative models and reflexive models (Brozoski and Bauer 2016; Galazyuk and Brozoski 2020). Interrogative models evaluate voluntary behavior (i.e., performing an action to obtain food when hearing a sound), while reflexive models evaluate involuntary behavioral responses to the acoustic startle reflex (Brozoski and Bauer 2016; Galazyuk and Brozoski 2020). Across species, the most frequently employed reflexive model uses the gap–prepulse inhibition of the acoustic startle (GPIAS) to determine the presence and course of tinnitus pathology (Galazyuk and Hebert 2015; Turner et al. 2006). To induce tinnitus, animals are either administered high doses of sodium salicylate (Su et al. 2012; Yang et al. 2007) or exposed to loud sound (Brozoski and Bauer 2016). In the latter case, animals under anesthesia are unilaterally exposed to loud broad-band noise while the contralateral ear is plugged to prevent hearing loss. In the GPIAS paradigm, acoustic startle responses are reduced, when a silent gap (e.g., 50 ms) is inserted before the startle sound (Smit et al. 2016). However, when a sound matching the tinnitus frequency is played and a silent gap is presented, the gap will not be perceived by the animal experiencing tinnitus, because it has been filled-in by the tinnitus frequency (Turner et al. 2006). Thus, animals experiencing tinnitus show increased startle responses in comparison to unexposed controls (Turner et al. 2006; Yang et al. 2007). Advantages of the GPIAS model are that it is relatively fast to administer, does not require training, and motivational states (i.e., frequently managed via diet restrictions) play a minor role (Brozoski and Bauer 2016). One of the disadvantages is habituation, i.e., when repeated, unconditioned reflexes diminish in amplitude (Lobarinas et al. 2013; Longenecker and Galazyuk 2011). This issue was addressed by administering fewer trials and by randomly varying the inter-stimulus interval (van Zwieten et al. 2019a, b). Based on the assumption that tinnitus pathogenesis relies on malfunctioning of a vast network of primary auditory and non-auditory structures (Llinas et al. 1999; Rauschecker et al. 2010), it has also been criticized that the GPIAS model does not take hyperacusis and emotional factors such as stress into account (Brozoski and Bauer 2016; Kleinjung and Langguth 2020). Thus, evaluating animal studies investigating MGB functioning in tinnitus pathology requires close monitoring of the paradigm choice, because even if motivational states do play a minor role in the GPIAS model, stress might still influence an animal’s performance.

### Animal studies investigating the MGB in tinnitus

Several studies investigated MGB changes in tinnitus animal models (Table 2).

**Table 2:** Animal studies investigating the MGB in tinnitus

Study	Subjects	Tinnitus induction	Tinnitus assessment	Paradigm	Method	Activity
Brozoski et al. 2012	10 Ctrl, 10 Tin	Unilateral NT (1 h, band-limited noise)	Interrogative model	In vitro–spectroscopy	Proton magnetic resonance spectroscopy	Decrease GABA and glu in contralateral MGB
Su et al. (2012)	6 Tin	Sodium salicylate	Reflexive model (GPIAS)	In vitro–single cell	Whole-cell patch-clamp	Decreased synaptic transmission (hyperpolarization resting membrane potential; decreased firing rates)
Kalappa et al. (2014)	9 Ctrl, 6 Tin	Unilateral NT(1 h, octave band noise)	Reflexive model (GPIAS)	In vivo (awake) –single cell	Tetrode microdrives	Increased spontaneous firing in Tin, mean bursts per minute, mean spikes per burst and mean burst duration
Sametsky et al. (2015)	10 Ctrl, 14 Tin, 4 non-Tin	Unilateral NT (1 h, octave band noise)	Reflexive model (PPI)	In vitro–single cell	Whole-cell patch-clamp	Increase in number of spikes per burst
Vianney-Rodrigues et al. (2019)	10 Tin	Sodium salicylate	None	In vivo (anesthetized) –LFP	Microelectrode arrays	Decrease theta, alpha, beta, increased coherence in gamma
Barry et al. (2019)	12 Ctrl, 16 Tin	Unilateral NT (2 h, pure tone)	Reflexive model (GPIAS, PPI)^a^	In vivo (anesthetized)–single cell	Microelectrodes	No differences for spontaneous firing rates between groups, increased bursting patterns in Tin, decreased percentages of spikes per burst in Tin
Van Zwieten et al. (2021)	5 Ctrl, 9 Tin	Unilateral NT (1.5 h octave band noise)	Reflexive model (GPIAS)^b^	In vivo (anesthetized)–single cell and LFP	Microelectrode and bipolar electrode	Decrease in fast responding neurons, increase in non-responsive neurons, increased spontaneous firing in neurons of sustained and suppressed type, fast responding neurons did not change the spontaneous firing rate, in both groups: DBS suppressed thalamocortical synchronization in beta and gamma bands

*Ctrl*, control animals, *GABA* gamma-aminobutyric acid, *GPIAS* gap–prepulse inhibition of the acoustic startle, *Glu* glutamate, *LFP* local field potential, *non-Tin* noise exposure but no tinnitus, *NT* noise trauma, *PPI* prepulse inhibition, *Tin* animals experiencing tinnitus

^a^Tinnitus was assessed using the GPIAS and the PPI model on a subgroup *n* = 9 from the *n* = 16 Tin rats

^b^The set-up did not allow valid discrimination between non-Tin and Tin animals

However, due to heterogeneous methodology and the overall limited number of studies, it is difficult to identify generalizable result patterns. When focusing on changes related to neurotransmitters, decreased GABA has been found in the MGB in rat models of tinnitus (Brozoski and Odintsov 2012; Llano et al. 2012). However, contradicting evidence exists (Sametsky et al. 2015). Administering high doses of sodium salicylate decreased the excitability of neurons in the MGB, leading to increased hyperpolarization of resting state potentials (Su et al. 2012; Wang et al. 2016). Another approach to assess alterations in the MGB in animals experiencing tinnitus is to investigate firing patterns, in vitro or in vivo in either anesthetized or awake animals. In vitro, both healthy control animals and rats with behavioral evidence of tinnitus, displayed burst firing after a current injection to the soma (Sametsky et al. 2015). Animals with tinnitus had an increased number of spikes per burst in comparison to controls and increased tonic GABAA currents. This suggests a shift towards increased tonic inhibition, which may result in abnormal bursting activity in the MGB, in turn leading to increased output from the MGB to higher auditory cortices (Sametsky et al. 2015). Moreover Sametsky et al. (2015) investigated whether changes in LTS responses could be associated to the increase of spikes per burst in rats with tinnitus. The authors found no differences between tinnitus and control animals in amplitude or area of LTS for bursts elicited by injecting a hyperpolarizing current. Thus, suggesting that multiple and additional mechanisms might play a role in the excitability of MGB neurons. Another study observed reduced numbers of neurons exhibiting burst activity patterns, decreased spikes per burst and bursts per minute in anesthetized rats who were administered an acoustic noise trauma, irrespective of tinnitus presence (Barry et al. 2019). Another study investigating the MGB in anesthetized rats with and without noise exposure classified four response types (i.e., fast, sustained, suppressed and no response) (van Zwieten et al. 2021). It was found that noise exposure resulted in an overall decrease of fast responding neurons, while non-responsive increased (van Zwieten et al. 2021). In addition, spontaneous firing rates increased in sustained and suppressed neurons, while this was not the case for fast responding neurons. Acquired LFPs suggest suppressed thalamocortical synchronization in the beta and gamma bands, independent of noise trauma (van Zwieten et al. 2021).

Oscillatory coherence between the MGB and the primary auditory cortex has been investigated using local field potentials (LFP) in anesthetized rats, while tinnitus was induced by sodium salicylate (Vianney-Rodrigues et al. 2019). Results indicate that sodium salicylate decreased theta, alpha, and beta oscillations in the MGB. Decreased coherence (i.e., the strength of a correlation between two signals as a function of frequency) between theta and alpha oscillations was further observed, while gamma coherence was increased between pairs of electrodes positioned in the MGB and PAC (Vianney-Rodrigues et al. 2019). Interestingly, when assessing the coherence (i.e., synchrony) between the MGB and PAC, sodium salicylate decreased coherence measures in the beta, alpha, and theta bands and again, enhanced coherence for the gamma band (Vianney-Rodrigues et al. 2019). Enhanced gamma coherence relates to previous research, as gamma band activity was suggested to be a direct neural correlate of tinnitus, influencing thalamo-cortical networks (Schlee et al. 2009; Sedley et al. 2012; van der Loo et al. 2009).

In awake rats, Kalappa et al. (2014) found similar results as Sametsky and colleagues (2015), confirming increased number of bursts per minute, increased mean burst duration and mean spikes in a burst. Kalappa et al. (2014) showed increased spontaneous firing in the MGD, MGM, and the MGV in a rat model of tinnitus. However, spontaneous firing rates in the MGB have also been found to be unaffected in rats with acoustic noise trauma or tinnitus (Barry et al. 2019). Most importantly, enhanced behavioral evidence of tinnitus pathology (i.e., increased z-scores of the raw-gap-startle in the GPIAs) was linked to higher spontaneous firing rates, irrespective of sound exposure (Kalappa et al. 2014). The increases in spontaneous firing could be specified by increases in bursts per minute, in mean spikes per burst, and in overall burst duration (Kalappa et al. 2014). Taken together, this suggests a shift towards a more spontaneous hyperactive bursting pattern of MGB neurons, moreover LFP studies show an altered coherence in the MGB in tinnitus.

### Human neuroimaging studies investigating the MGB in tinnitus

To the best of our current knowledge, intracranial or single-unit recordings from the MGB in humans do not exist. Paradigms investigating functionalities of the MGB in persons with tinnitus therefore often rely on measures of functional and structural connectivity obtained with fMRI (Table 3).

**Table 3:** Human studies reporting effects on the auditory thalamus in tinnitus

Study	Participants		Tinnitus duration	Tinnitus assessment	Control for HL	Paradigm	Method	Results auditory thalamus
	Ctrl	Tin						
*Structural MRI*								
M*ü*lhau et al. (2006)	28	28	> 4.5y	GHS	Yes^a^	Structural lesions	MRI	Increased gray matter concentration in the MGB in tinnitus
Sugiua et al. (2008)	1450	743	NA	Questionnaire	No	Structural lesions	MRI	Inverse association between cerebral infarction and tinnitus
*Functional MRI*								
Smits et al. (2007)	10	7 BLTin, 22 LTin, 13 RTin	> 5y	Pitch matching	No	Task-based	fMRI	Symmetrical signal change in BLTin, Ltin decreased activation contralateral to Tin
Chen et al. (2014)	32	32	> 3.4y	THQ	Yes	Resting-state	fMRI (ALFF)	Decreased activity in bilateral thalamus
Zhang et al. (2015)	33	31	> 3.5y	THQ	Yes^a^	Resting-state	fMRI (VBM)	LThal = decrease in: MTG, mOFC, mFG,R PrecG, calcarine cortex; increase: angular gyrus, mCC, postCB; RThal = decrease in: STG, amygdala, SFG, L PrecG, mOG, increase: pCC, postCB; no changes in thalamic volume
Allan et al. (2016)	55	73	min. 6 months	THI or THQ	Yes^b^	Resting-state	fMRI (VBM, SBM)	Reduced white matter volume in the right MGB in severely affected tinnitus subgroup, reduced gray matter volume with increasing HL in the bilateral MGB; when comparing the subgroup Tin no HL vs HC, no effects in the MGB were found
Hofmeier et al. (2018)	17	17	> 4 weeks	GHS	Yes^a^	Resting-state/task-based	fMRI	Reduced connectivity in the left MGB in tinnitus, reduced sound-evoked response in the MGB in tinnitus
Han et al. (2019)	27	27	≥ 6 months	THI	Yes^a^	Resting-state	fMRI (FCS)	Increased functional connectivity strength in the thalamus tinnitus compared to controls
Lv et al. (2020)	25	25	> 2y	THI	Yes^a^	Resting-state	fMRI	Increased connectivity between thalamus and IFG and ACC at baseline
Berlot et al. (2020)	6	6	> 0.5y	TQ	Yes	Resting-state/task-based	fMRI	Decreased connectivity starting at the MGB to higher auditory cortices
*DTI*								
Aldhafeeri et al. (2012)	14	14	> 6y	THI	Yes	Resting-state	DTI	Decreased white matter integrity in persons with tinnitus in the anterior thalamic radiation
Benson et al. (2014)	13 NIHL	13 NIHL Tin	min. 6 months	THI	Yes	Resting-state	DTI	Four clusters in the anterior thalamic radiation reflected increased white matter integrity for NIHL Tin
Gunbey et al. (2017)	20	18 TinHL, 18 Tin	> 4y	THI, VAS	Yes	Resting-state	DTI	Decreased connectivity in MGB in Tin patients
*ECoG*								
De Ridder et al. (2011)	–	1 BLTin	14y	VAS	No	Awake –resting-state	ECoG	Tinnitus-linked gamma-theta coupling, hypothesized to be influenced in thalamus
Sedley et al. (2015)	–	1 BLTin	Approx. 15y	THI	No	Awake–task-based	ECoG	Tinnitus-linked delta oscillations, hypothesized to be triggered in thalamus

*ACC* anterior cingulate gyrus, *ALFF* amplitude low-frequency fluctuations, *BLTin* bilateral tinnitus, *Ctrl*, Controls, *DTI* diffusion tensor imaging, *ECoG* electrocorticography, *FCS* functional connectivity strength, *fMRI*, functional magnetic resonance imaging, *GHS* Goebel–Hiller-Score tinnitus questionnaire, *HC* healthy controls, *HL* hearing loss, *HQ* hyperacusis questionnaire, *IFG* inferior frontal gyrus, *LFP* local field potentials, *LTin* left tinnitus, *mCC* medial cingulate cortex, *mFG* middle frontal gyrus, *MGB* medial geniculate body, *mOFC* medial orbitofrontal cortex, *mOG* middle occipital gyrus, *MTG* middle temporal gyrus, *MRI* magnetic resonance imaging, *NIHL* noise-induced hearing loss, *postCB* posterior cerebellum, *PrecG* precentral gyrus, *RTin* right tinnitus, *SBM* surface-based morphometry, *STG* superior temporal gyrus, *THI* tinnitus handicap inventory, *Tin* tinnitus, *TinHL* tinnitus with hearing loss, *THQ* tinnitus handicap questionnaire, *TQ* tinnitus questionnaire, *VAS* visual analog scale, *VBM* voxel-based morphometry

^a^In addition, controlled for hyperacusis

^b^Forming subgroups from the original sample

A large cross sectional population-based study conducted in Japan identified an inverse relation between cerebral infarction in the thalamus and tinnitus, which could either be interpreted as cerebral infarctions inhibiting tinnitus or as increased tinnitus symptoms being present with no or reduced cerebral infarctions (Sugiura et al. 2008). Investigations of MGB volume in persons with tinnitus is generally in favor of similar MGB sizes in persons with tinnitus and controls (Landgrebe et al. 2009; Zhang et al. 2015), but opposing findings exist (Allan et al. 2016; Muhlau et al. 2006; Tae et al. 2018). Irrespective of hearing loss, diffusion tensor imaging (DTI) revealed that MGB connectivity was bilaterally reduced in persons with tinnitus (Gunbey et al. 2017). Another DTI study confirmed reduced white matter integrity in persons with tinnitus in the anterior thalamic radiation (Aldhafeeri et al. 2012), but opposing evidence exists, suggesting increased white matter integrity in the anterior thalamic radiation in persons experiencing tinnitus after noise induced hearing loss (Benson et al. 2014). A task-based fMRI study investigated a group of persons with chronic tinnitus listening to music segments (Smits et al. 2007). When participants experienced bilateral tinnitus, signal change in the MGB was bilateral and if participants experienced tinnitus in the left ear, the right thalamus had a lower activation ratio (Smits et al. 2007). The reverse pattern (i.e., right tinnitus percept) was not significant, which is likely attributable to a smaller sample size. Another task-based fMRI study suggests reduced sound-evoked responses in the MGB in persons with tinnitus (Hofmeier et al. 2018).

Resting-state fMRI in persons with tinnitus suggests overall decreased functional connectivity between the MGB and cortical regions. Han et al. (2019) found increased functional connectivity strength in the thalamus in persons with tinnitus compared to controls. Amplitude low-frequency fluctuations (ALFFs), a measure that has previously been related to spontaneous neural activity (Lv et al. 2018), was bilaterally decreased in the thalamus in persons with chronic tinnitus (Chen et al. 2014). There was a positive correlation between tinnitus duration and increases in ALFFs in the superior frontal gyrus (SFG) (Chen et al. 2014). Decreased functional connectivity between the left thalamus and right middle temporal gyrus (MTG), right middle OFC, left middle frontal cortex, right precentral gyrus was found in persons with chronic tinnitus (Zhang et al. 2015). When the right thalamus was used as a seed region, decreased functional connectivity between the right thalamus and the left superior temporal gyrus (STG), left amygdala, right SFG, left precentral gyrus, and left middle occipital gyrus was observed (Zhang et al. 2015). Conversely, increases in functional connectivity were observed between the thalamus and the posterior cerebellum, middle, and posterior cingulate cortices (Zhang et al. 2015). Taken together, these results confirm that the thalamus plays a central role in a wider thalamo-cortical network implicated in tinnitus pathology. However, Zhang et al. (2015) and Chen et al. (2014) did not differentiate between subcomponents within the thalamus (i.e., parts of the MGB), and it is noteworthy that decreased functional connectivity and increased spontaneous neural activity between the thalamus and SFG were observed in both experiments. Lv et al. (2020) investigated changes in functional connectivity before and after sound therapy. This study found higher connectivity measures at baseline for the tinnitus group between the thalamus, the inferior frontal gyrus (IFG; Brodman area (BA) 45), and the anterior cingulate cortex (ACC; BA 33), which were restored (i.e., decreased) after treatment (Lv et al. 2020). Reduced tinnitus severity could be associated with decreased functional connectivity between the right thalamus and the right IFG. Thus, the study of Lv et al. (2020) indicates increased functional connectivity for persons with tinnitus at baseline, whereas, a different pattern (i.e., decrease in functional connectivity) for the superior and middle frontal gyrus was previously suggested by Zhang et al. (2015). Nevertheless, Lv et al. (2020) suggests that decreased functional connectivity may represent a decrease in attention in tinnitus pathology and a reduction in the involvement of the noise cancellation system (i.e., sensory gating) (Rauschecker et al. 2010), which supports the previously discussed findings. Another recent study focused on resting-state activity in persons with tinnitus (Berlot et al. 2020). Here, the MGB seed regions were chosen based on responses to the individual tinnitus frequency and to a control frequency, which had the farthest distance to the tinnitus pitch (i.e., using tonotopic maps from each participant-control pair), while connectivity was measured along several centers of the auditory pathway. Results suggest reduced connectivity measures in persons with tinnitus starting at the level of the MGB (Berlot et al. 2020). Thus, in persons with tinnitus functional connectivity between the MGB and the primary auditory cortex and between the primary and the secondary cortices were reduced for the tinnitus and the control frequency seed (Berlot et al. 2020). These findings are in line with the findings reported by Zhang et al. (2015), suggesting reduced connectivity between the left thalamus seed and the right MTG.

Previously the inhibitory influence of the TRN on the MGB was incorporated in the noise-cancellation approach, stating that in persons without tinnitus, the TRN cancels out or filters unwanted sounds (Leaver et al. 2011; Rauschecker et al. 2010; Zhang 2013). However, in persons with tinnitus, this filtering becomes distorted, leading to the perception of tinnitus (Leaver et al. 2011; Rauschecker et al. 2010; Zhang 2013). To the best of our knowledge, there is only one human study investigating the TRN in tinnitus (Gunbey et al. 2017). Results by Gunbey et al. (2017) for the TRN parallel their findings for the MGB in persons with tinnitus this is reflected in decreased fractional anisotropy (FA) and increased apparent diffusion coefficient (ADC) values.

**Fig. 2:**
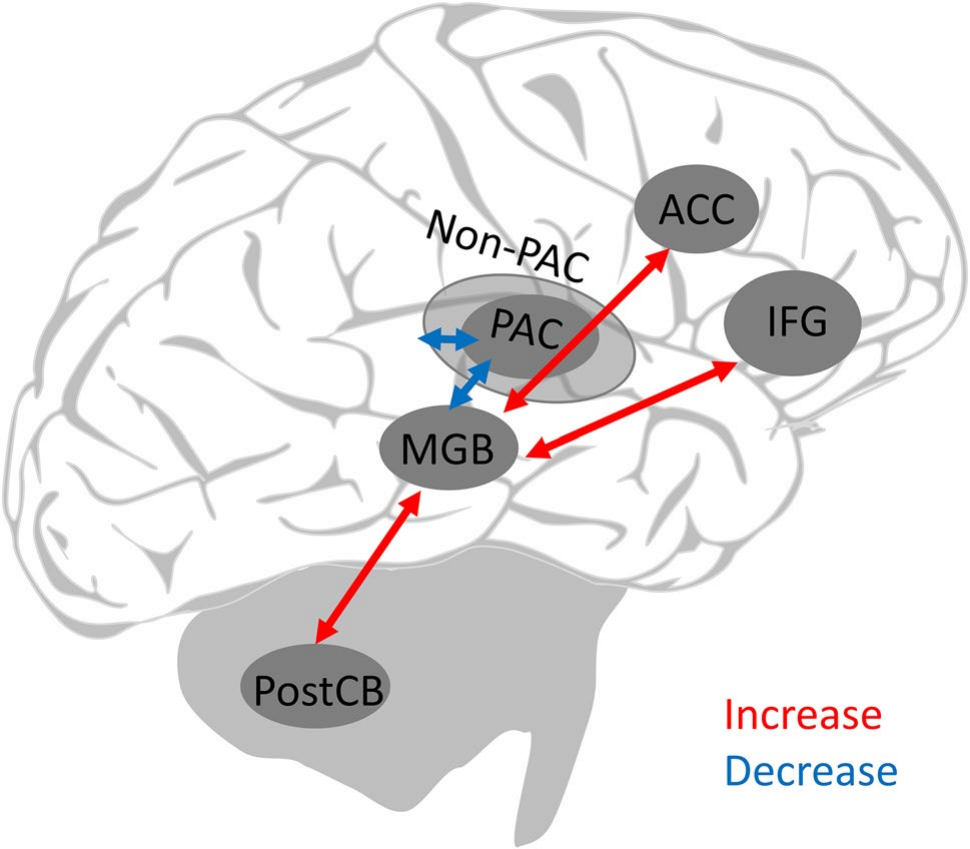
Summary and schematic representation of increased/decreased functional connectivity measures between the MGB and cortical areas. The representation is based on baseline measures of Lv et al. (2020), Berlot et al. (2020), and Zhang et al. (2015). Depicted are only areas with altered connections to the bilateral MGB. Zhang et al. (2015) observed decreased connectivity between the left thalamus to the medial frontal gyrus and the right thalamus and superior frontal gyrus, contrasting with increased connectivity between the MGB and the IFG (BA 45) by Lv et al. (2020). Zhang et al. (2015) further observed increased connectivity between the left thalamus and the middle cingulate cortex, and the right thalamus and the posterior cingulate cortex. ACC, Anterior cingulate cortex (BA 33), PAC, Primary auditory cortex, Non-PAC, Non-Primary auditory cortices, IFG, Inferior frontal gyrus, MGB, Medial geniculate body, PostCB, Posterior cerebellum. Lv et al. (2020), Berlot et al. (2020), Zhang et al. (2015)

From the existing evidence, it can hence be concluded that functional connectivity between the auditory thalamus and auditory cortices (i.e., PAC, non-PAC) seems to be reduced in persons with tinnitus. In addition, there is increased connectivity between cingulate cortices, likely the IFG and posterior cerebellum, which indicates changes in the function of a widespread network due to disrupted thalamo-cortical functional connectivity (Fig. 2). A recent study by Lin et al. (2020) compared topological network changes in gray matter between persons with tinnitus and controls using a graph-theoretical approach. Their betweenness centrality analyses revealed exclusive hubs in the amygdala and parahippocampus in persons with tinnitus, while hubs in the auditory cortex, insula, and the thalamus were exclusively present in controls but not in persons with tinnitus (Lin et al. 2020). The absence of the thalamus hub in the tinnitus group suggests altered interactions between the auditory thalamus and related auditory regions.

Currently, there are no invasive human MGB recordings available, but a limited amount of case studies performed intracranial cortical recordings in patients that also experienced tinnitus, which can be linked to alterations in the MGB. Two case studies investigated persons with tinnitus, while performing intracranial recordings from the (secondary) auditory cortex (i.e., electrocorticography, ECoG). Results from one study in a person suffering from severe tinnitus for 14 years showed increased gamma and theta activity in one of the eight implanted electrode poles (De Ridder et al. 2011). Interestingly, the pole reflecting the enhanced gamma-theta coupling was located in an area that showed maximal BOLD activity levels in response to tones in the tinnitus frequency during an fMRI session (De Ridder et al. 2011). Lastly, intracranial measures recorded from the auditory cortex in a patient suffering from complex temporal lobe seizures suggest that tinnitus suppression (measured via residual inhibition) is linked to widespread delta band coherence (Sedley et al. 2015). Moreover, Sedley et al. (2015) observed increases in gamma (> 28 Hz) and beta2 (20–28 Hz) bands during tinnitus suppression. The authors identified three tinnitus sub-networks. The first is the large tinnitus-driven network characterized by changes in delta coherence in addition to delta, theta and alpha power changes. The second is the tinnitus memory network involved in auditory memory and mainly characterized by increases in alpha power. The third network is the tinnitus perception network characterized by changes in the gamma and beta range (Sedley et al. 2015). Although these networks do not specifically focus on the functioning of the MGB, the authors note that the observed alterations in delta oscillations may be triggered by the thalamus.

## Overarching framework: linking animal and human findings and implementation in theoretical framework of temporal predictions

Due to fundamental methodological differences, the integration of results from animal studies investigating tinnitus and MGB functioning in humans faces several issues (for a summary of animal and human studies (Tables 1, 2). It is therefore important to evaluate the advantages and constraints of each method in order to draw conclusions about how to relate measurements at different functional levels to each other. Animal studies employ methods measuring single cell and multi-unit activity, or LFPs. These approaches allow drawing conclusions about neurotransmission, spontaneous firing rates, or coherence. In contrast, human studies report data obtained from large populations of neurons, or even whole brain analyses, with the thalamus increasingly being recognized as a seed region for connectivity analyses (Berlot et al. 2020; Lv et al. 2020; Zhang et al. 2015). As the MGB is a small subcortical structure, accessibility by means of high-temporal neuroimaging methods such as EEG/MEG to assess neural synchrony and coherence is severely limited. Therefore, resting-state functional connectivity or structural measurements are most common. Next to these methodological constraints, variability in tinnitus pathology is another critical factor. In animals, tinnitus is often induced using noise trauma or by administering sodium salicylate before behaviorally testing for tinnitus using either interrogative or reflexive models (i.e., GPIAS). While sodium salicylate was found to reliably induce tinnitus (Day et al. 1989; Lobarinas et al. 2004; Stolzberg et al. 2012; Su et al. 2012), affective components such as anxiety or stress are typically not considered (Brozoski and Bauer 2016; Kleinjung and Langguth 2020). In addition, the type of tinnitus induced with sodium salicylate is quite different when compared to the poise induced model, as tinnitus experience after receiving sodium salicylate is more intense and not accompanied by hearing loss, which could occur when administering a noise trauma (Norena et al. 2010). The GPIAS model on the other hand has been criticized to not be transferable to humans, because in humans gap detection thresholds were similar for persons with tinnitus and controls (Clayton and Koops 2021; Zeng et al. 2020). In humans, tinnitus pathology is heterogeneous as well (Cederroth et al. 2019; Kleinjung and Langguth 2020). For instance, persons with tinnitus differ with respect to perceptual characteristics, time course, comorbidities and response to interventions (Kleinjung and Langguth 2020). The identification of reliable tinnitus subtypes therefore remains a major challenge (Cederroth et al. 2019; Kleinjung and Langguth 2020). In general, tinnitus is likely preceded by peripheral hearing loss and the majority of persons with tinnitus have abnormal audiograms. However, several issues remain, as for example, the majority of people experiencing hearing loss does not develop tinnitus (Roberts et al. 2006; Sedley 2019). Of note that peripheral hearing loss leads to deafferentiation at the level of the cochlear, but that even without behaviorally measurable hearing loss, deafferentaition is probably still present in persons with tinnitus (Weisz et al. 2006). Another unresolved paradox is that the development of tinnitus is difficult to explain by either a pure peripheral or central model, although, even though tinnitus is thought to be initialized by peripheral hearing loss (Sedley et al. 2016). Therefore, in order to bridge the gap between the results obtained by animal models and human studies, additional research is clearly needed to link the underlying mechanisms to the known functional characteristics of the auditory thalamus.

### Thalamo-cortical dysrhythmia and sensory gating in tinnitus

Several theoretical approaches attempted to explain the development of tinnitus (for an overview see: Sedley et al. (2016)). However, only a few specifically account for MGB function. The noise cancellation approach for instance, proposes interactions between limbic structures and the auditory thalamus in tinnitus pathogenesis in a top–down fashion (Rauschecker et al. 2010; Song et al. 2015). Healthy individuals engage the non-classical auditory pathway to evaluate the emotional content of sound stimuli in parallel to auditory processing along the classical auditory pathway. Unpleasant auditory input is normally “cancelled out” at the level of the MGB (Rauschecker et al. 2010). In persons with tinnitus, however, the noise cancellation (i.e., sensory gating) mechanism is dysfunctional, leading to disinhibition of the MGB, possibly contributing to the perception of a tinnitus sound (Elgoyhen et al. 2015). Sensory gating may also be conceived as an adaptive mechanism that is employed to filter out irrelevant information based on spectral and temporal information to predictively adapt and optimize auditory function (Schwartze and Kotz 2013).

Another approach suggests that distorted firing patterns and altered oscillatory coupling mechanisms at the level of the MGB may induce tinnitus in a bottom–up fashion (De Ridder et al. 2015; Llinas et al. 1999). The thalamo-cortical dysrhythmia hypothesis suggests aberrant neural synchrony within and between the thalamus and cortex. Decreased auditory input leads to altered rhythmic burst firing in the MGB (i.e., increased low-frequency thalamic oscillations, triggered by LTS), which leads to increased activation in higher auditory cortices in theta, delta and gamma ranges (De Ridder et al. 2015; Llinas et al. 1999). De Ridder et al. (2015) speculate that in tinnitus with limited deafferentiation, alpha oscillations slow down and turn into theta oscillations, which are coupled to gamma oscillations, while gamma has been interpreted as the bottom–up transmitted prediction error. In severe deafferentiation, however, auditory information retrieval might be mediated by parahippocampal auditory memories acting in the theta range (De Ridder et al. 2015). Altered high frequency activity in the dorsal ACC or pregenual anterior cingulate might represent allostasis processes involved in a reference resetting, indicating that the new norm state might be the tinnitus state and not the silent state (De Ridder et al. 2015). Theta is suggested to act as a carrier frequency, needed to activate the tinnitus network, while gamma encodes the tinnitus intensity (De Ridder et al. 2011, 2015).

Support for dysfunctional sensory gating mechanisms in the thalamus in persons with tinnitus was recently provided by Lin et al. (2020), showing in a graph-theoretical approach that the thalamus hub was only present in the control group and not in persons with tinnitus. Eliciting tinnitus-like symptoms using an auditory illusion in healthy young adults without hearing loss, resulted in enhanced total theta power in the parahippocampus, pregenual ACC, the ventro-medial PFC and OFC, further supporting inadequate sensory gating even in healthy participants (Mohan et al. 2020). The concept of sensory gating allows linking the intrinsic firing modes of the thalamus, the top–down noise-cancellation approach (Rauschecker et al. 2010) and the bottom–up thalamo-cortical dysrhythmia approach (De Ridder et al. 2015; Llinas et al. 1999) into a common theoretical framework for predictive adaptation.

**Fig. 3:**
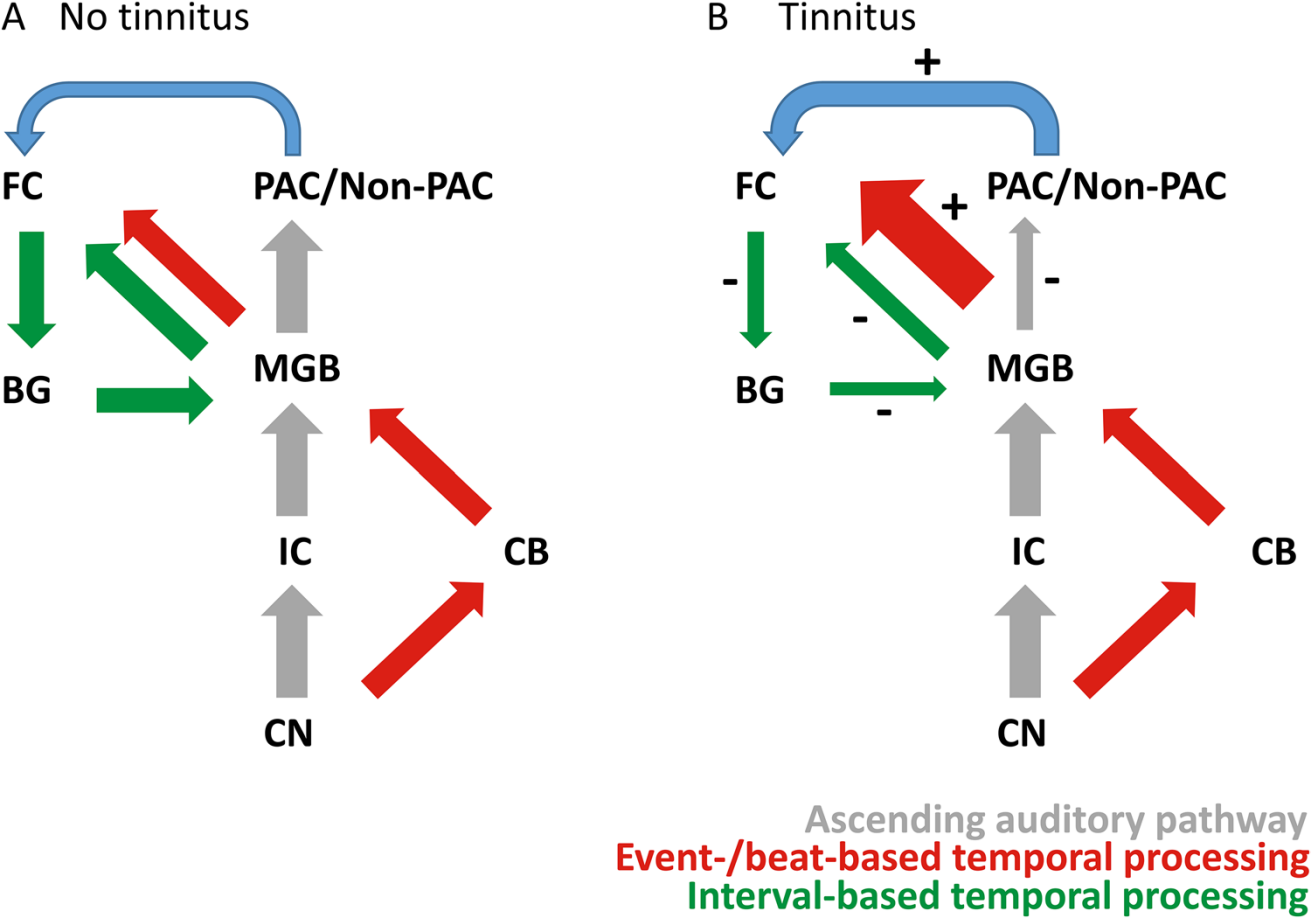
Schematic representation of the neural architecture for specific temporal prediction in persons without tinnitus a and with tinnitus b. Here, the ascending auditory pathway does not distinguish between the classical and the non-classical auditory pathway. The schema does not depict predictive top–down modulation of the network by dynamic input. The MGB forms a major hub in transmitting a timing signal to higher cortical areas (event-/beat-based temporal processing (red)). This signal forms the basis for interval-based temporal processing (green) in BG circuits. Parallel activation and integration of memory representations recruit connections between temporal and frontal cortices (blue). In tinnitus (B), connections between the MGB and auditory cortices are reduced. Starting from the MGB, increased burst and spontaneous firing leads to an increase in event-/beat-based temporal processing. Tonic firing is proposed to be reduced, reflected by decreased interval-based temporal processing, as depicted by the different arrow sizes + and – signs. In severe deafferentiation, memory retrieval increasingly relies on parahippocampal and auditory areas. *PAC*/*Non-PAC* primary and non-primary auditory cortices, *BG* basal ganglia, *CB* cerebellum, *CN* cochlear nucleus, *FC* frontal cortex, *IC* inferior colliculus, *MGB* medial geniculate body

The functional principle of sensory gating (i.e., the filtering out of irrelevant information) has been associated with reduced neural activity for predicted information (i.e., gating out) and increased activity for unpredicted information (i.e., gating in) (Grunwald et al. 2003; Marshall et al. 2004; Pratt et al. 2008; Schwartze and Kotz 2013). Schwartze and Kotz (2013) introduced an integrative subcortico-cortical network for feature-based and temporal predictions. Feature-based information (used to generate “what” predictions based on the formal structure of a dynamic input) is primarily encoded linearly (i.e., engaging thalamic tonic firing), whereas temporal information (used to generate “when” predictions based on salient input features such as onsets, offsets, and rising energy contours) are encoded non-linearly (i.e., engaging thalamic burst firing) in the MGB. The resulting dual-pathway neural architecture for specific temporal prediction may provide a common framework for understanding how alterations in the MGB could translate to the experience of tinnitus (Fig. 3). Reduced sensory gating (i.e., reduced inhibition) at the level of the cortex, as suggested by the noise-cancellation approach, the thalamo-cortical dysrhythmia approach and by the increases in spontaneous firing rates at the level of the MGB may be key to guide understanding of the role of the MGB in tinnitus pathology.

### Sensory gating in the MGB and temporal stimulus predictability

Tinnitus has previously been associated with predictive coding (De Ridder et al. 2015; Hullfish et al. 2019; Sedley et al. 2016). (Latent) prediction errors are likely represented by gamma oscillations. Attention, memory and learning towards the tinnitus experience might modulate the influence of prediction errors at higher functional levels (Sedley et al. 2016). Sedley and colleagues suggest that high frequency gamma oscillations convey bottom–up prediction errors that are compared to top–down predictions, involving lower frequency beta oscillations (Sedley et al. 2016). Low-frequency oscillations, especially in the theta range, have been suggested to function as carriers, while being able to modulate high-frequency oscillations (Canolty et al. 2006). Hullfish et al. (2019) further suggest that differential predictive mechanisms might underlie acute or chronic tinnitus. Moreover, increased mismatch negativity responses (MMN) were observed in persons experiencing tinnitus, indicating violated sensory predictions (Sedley et al. 2019). However, it may still be necessary to further differentiate temporal and formal aspects of predictions in relation to MGB functioning. Here we suggest that alterations in the thalamic firing modes, likely caused by tinnitus, contribute to the observed changes in oscillatory activity in higher cortical auditory areas. As it is proposed that sensory gating (e.g., gating out the predicted stimuli in a paired-stimulus paradigm) is dysfunctional in tinnitus (Bayazitov et al. 2013; Lin et al. 2020), we propose that sensory gating at the level of the MGB can be differentially influenced by altering the temporal and formal predictability of the input signal (Fig. 3).

It is possible that there is a direct input route for auditory sensory processing to the cerebellum, as suggested by an ALE meta-analysis by Petacchi et al. (2005). In addition, research in the cat auditory system supports direct connections between the cochlear nucleus and the cerebellum (Huang and Burkard 1986; Huang et al. 1982). Rapid cerebellar transmission is suggested to encode event-based temporal information (Schwartze and Kotz 2013; Teki et al. 2011a, b), triggering a burst firing mode (i.e., non-linear) in the thalamus (Fig. 3a). In other words, the cerebellum receives auditory input and transmits successive events via the thalamus to frontal areas, mimicking a “clock signal”. The basal ganglia encodes the relation (i.e., the interval-based timing) between events and feeds this information back to frontal areas (Allman and Meck 2012; Schwartze and Kotz 2013; Teki et al. 2011a, b). The auditory cortex is connected to frontal cortices, while receiving input from parahippocampal areas for memory retrieval, as suggested by De Ridder et al. (2015) and Schwartze and Kotz (2013). Moreover, the frontal cortex feeds information about stimulus identity and interval duration back to the basal ganglia (Matell et al. 2005). In persons with tinnitus however, signal encoding is less efficient. Animal and human studies have shown altered connectivity between the MGB and cortical areas, and increased bursting and spontaneous firing rates in the MGB itself, leading to less precise predictive adaptation. The connectivity between the MGB and the primary and secondary auditory cortex is probably reduced (Fig. 3b). In addition, as increased bursting in the MGB has been observed in persons with tinnitus, increased event- or beat-based processing may be observed starting from the thalamus, while the processing interval-based durations might be reduced. Especially in severely affected persons, memory retrieval from parahippocampal areas probably strengthens the association between the auditory cortices and frontal areas. Manifestations of the described alterations in this neural architecture for specific temporal predictions might be observed by increases in gamma and slow frequency bands such as theta or delta, as suggested by work from Sedley and colleagues or De Ridder et al. (2015) and by dysfunctional sensory gating mechanisms in persons with tinnitus (for example: Schwartze et al. (2011, 2013). However, it still needs to be elucidated if it is possible to influence and eventually optimize synchronization between the thalamus and the auditory cortices, to ultimately compensate for the thalamo-cortical dysrhythmia by altering the rhythmical structure of the input signal. Compensation of the thalamo-cortical dysrhythmia would allow treating tinnitus at the level of the MGB and to reinstate its functionality.

## Conclusion

Based on the limited number of studies investigating MGB functioning in tinnitus pathology and their overall heterogeneous approaches, it can be concluded that tinnitus is associated with increased spontaneous firing in the MGB, decreased functional connectivity between the MGB and a widespread thalamo-cortical network, in addition to decreased connectivity between the MGB and auditory cortices. Decreased functional connectivity between the MGB and auditory cortices can lead to reduced inhibition at the level of the auditory cortex. Parallel increased functional connectivity between the ACC and the IFG and the MGB may represent dysfunctional attentional processes or allostatic mechanisms. Similarly, altered patterns of oscillatory activity have been observed between the MGB and cortical areas, mainly expressed as increased activity in high-frequency gamma and beta bands, decreased activity in delta bands, and altered theta and alpha coherence, providing support for the thalamo-cortical dysrhythmia hypothesis. However, the existence and contribution of several local sub-networks to the development and maintenance of tinnitus, as suggested by Sedley et al. (2015), should not be neglected. Here, we link changes in thalamic firing modes and oscillatory bands to tinnitus. We suggest that these changes modulate the function within a neural architecture mediating predictive adaptation of an organism to the auditory environment. Modulation of temporal characteristics of input signals might influence this neural architecture for predictive adaptation, likely altering the tinnitus experience. Therefore, modulation of temporal characteristics could ultimately help establish new directions for treatment options for persons with tinnitus.

## Data Availability

Not applicable.
